# The Role of Glutathione in Selected Viral Diseases

**DOI:** 10.3390/antiox12071325

**Published:** 2023-06-22

**Authors:** Joanna Wróblewska, Marcin Wróblewski, Iga Hołyńska-Iwan, Martyna Modrzejewska, Jarosław Nuszkiewicz, Weronika Wróblewska, Alina Woźniak

**Affiliations:** 1 Department of Medical Biology and Biochemistry, Ludwik Rydygier Collegium Medicum in Bydgoszcz, Nicolaus Copernicus University in Torun, 87-100 Torun, Poland; joanna.wroblewska@cm.umk.pl (J.W.); marcin.wroblewski@cm.umk.pl (M.W.); martyna.modrzejewska@cm.umk.pl (M.M.); jnuszkiewicz@cm.umk.pl (J.N.); 2 Department of Pathobiochemistry and Clinical Chemistry, Faculty of Pharmacy, Ludwik Rydygier Collegium Medicum in Bydgoszcz, Nicolaus Copernicus University in Torun, 87-100 Torun, Poland; igaholynska@cm.umk.pl; 3 Students Research Club of Medical Biology, Department of Medical Biology and Biochemistry, Faculty of Medicine, Ludwik Rydygier Collegium Medicum in Bydgoszcz, Nicolaus Copernicus University in Torun, 87-100 Torun, Poland; 316714@stud.umk.pl

**Keywords:** glutathione, viral hepatitis, hepatitis A virus, hepatitis B virus, hepatitis C virus, hepatitis E virus, liver, glutathione peroxidase, glutathione reductase, glutathione-S-transferase

## Abstract

During inflammatory processes, immunocompetent cells are exposed to substantial amounts of free radicals and toxic compounds. Glutathione is a cysteine-containing tripeptide that is an important and ubiquitous antioxidant molecule produced in human organs. The intracellular content of GSH regulates the detoxifying capacity of cells, as well as the inflammatory and immune response. GSH is particularly important in the liver, where it serves as the major non-protein thiol involved in cellular antioxidant defense. There are numerous causes of hepatitis. The inflammation of the liver can be caused by a variety of infectious viruses. The relationship between oxidative stress and the hepatitis A virus (HAV), hepatitis B virus (HBV), hepatitis C virus (HCV), and hepatitis E virus (HEV) infection is not fully known. The aim of this study was to examine the relationship between hepatotropic viruses and glutathione status, including reduced glutathione (GSH) and oxidized glutathione (GSSG), as well as antioxidant enzymes, e.g., glutathione peroxidase (GPx), glutathione reductase (GR) and glutathione-S-transferase (GST) in liver diseases.

## 1. Introduction

Viral hepatitis is a significant global public health concern. Each year, 1.4 million individuals succumb to cirrhosis and liver cancer related to viral hepatitis [1]. Regrettably, the majority of the infected population remains unaware of their condition [2]. Despite the implementation of infection control measures over the several past decades, eradication or substantial disease reduction remains elusive [1]. Viral hepatitis is caused by a variety of unrelated hepatotropic viruses, such as the hepatitis A virus (HAV), hepatitis B virus (HBV), hepatitis C virus (HCV), hepatitis D virus (HDV), and hepatitis E virus (HEV) [3]. Additionally, numerous non-hepatotropic viruses can lead to liver dysfunction. This group of viruses includes human herpesviruses, such as the Epstein–Barr virus; cytomegalovirus; herpes simplex virus-1 (HSV-1) and -2; varicella-zoster virus; human herpesvirus-6, -7, and -8; parvovirus B19; rubella virus; measles virus; Zika virus; dengue virus; and severe acute respiratory syndrome coronavirus 2 [4,5,6,7].

In recent years, the role of oxidative stress in diseases of viral etiology has increasingly been indicated [8]. Oxygen consumption, which is essential for life, contributes to the generation of reactive oxygen species (ROS) within cells [9]. These ROS, generated under physiological conditions, are a natural byproduct of cellular metabolism. The mitochondrial electron transport chain plays a significant role in ROS generation, with mitochondria and NADPH oxidases (NOXs) serving as the primary cellular sources [10]. The liver, an organ with a high concentration of mitochondria, is particularly involved in ROS production [11]. ROS encompass both free oxygen radicals and non-radical species, including major molecules, such as superoxide anion radical (O_2_^•−^), hydrogen peroxide (H_2_O_2_), hydroxyl radical (^•^OH), and nitric oxide (^•^NO), with the latter being classified as a reactive nitrogen species [12]. Oxidative stress occurs when the balance between ROS generation and antioxidant defense mechanisms is disrupted in favor of oxidants [13]. This imbalance can potentially lead to molecular-level cell damage, including the oxidation of proteins, DNA, and lipids [14]. Prolonged oxidative stress has been linked to various pathological conditions, such as chronic inflammation, neurodegenerative diseases, cardiovascular disorders, and cancer [15,16,17]. Antioxidant enzymes, such as superoxide dismutases, catalase, and glutathione peroxidases (GPx) are essential for maintaining the delicate balance between ROS production and scavenging, thus protecting cells from the harmful effects of oxidative stress [18]. In addition to endogenous antioxidant defenses, exogenous antioxidants found in various foods, such as polyphenols, vitamin C, vitamin E and carotenoids, can also help neutralize ROS and support cellular health [19]. Viral infections may lead to elevated levels of ROS and weakened antioxidant defenses, resulting in oxidative stress [20]. Understanding the role of oxidative stress in the context of viral infections may provide valuable insights into potential therapeutic strategies and interventions for mitigating virus-induced cellular damage. Among non-enzymatic antioxidants, reduced glutathione (GSH) plays a particularly important role in ensuring the redox balance [21]. Research results indicate that GSH and oxidized glutathione—glutathione disulfide (GSSG)—may affect the course of viral liver diseases [22,23]. This publication offers an extensive analysis of the latest scientific literature sourced from multiple databases, such as PubMed, Google Scholar, and Web of Science. The aim of this review article is to investigate the recent studies on the roles of oxidative stress and GSH as molecules with an antioxidant potential in the development and progression of viral liver diseases.

## 2. Glutathione and Its Antioxidant Properties and Implications for Health

The tripeptide γ-l-glutamyl-l-cysteinyl-glycine, known as glutathione, is the main antioxidant in aerobic cells [24,25] and is considered the most prevalent non-protein thiol in mammalian cells [26]. Its intracellular concentration equals from 1 to 15 mM [27,28]. It occurs in the form of GSH and GSSG [29]. Glutathione is generated in the process of synthesis as a reduced form, which can generate GSSG, with which it equilibrates and determines the redox environment [30].

GSH is synthesized in the cytoplasm of animal cells from glutamic acid, cysteine, and glycine [31], with the participation of two enzymes: glutamate–cysteine ligase and glutathione synthetase [32]. Simultaneously, the degradation of GSH to its constituent amino acids may occur with the participation of a γ-glutamyl cyclotransferase, among others [33]. Part of the generated GSH, depending on the needs, can be transported from the cytoplasm to cellular organelles, e.g., the cell nucleus, mitochondrion, endoplasmic reticulum, or GSH may also be effluxed out of the cell into the extracellular medium [34]. An extracellular pool of GSH may also be used for transport between different organs [34]. The major producer and exporter of GSH is the liver [35]. GSH may also be of exogenous origin. Contained in the diet, it may be partly absorbed from the small intestine [36]. De novo synthesis of GSH is controlled by the nuclear factor erythroid 2-related factor 2 (Nrf2), as it is a major regulator of oxidative stress signaling [37,38]. This transcription factor participates in the protection of cells against oxidative stress by upregulating the expression, among others, of genes involved in the antioxidant GSH pathway [39,40]. Among the factors activating the pathway regulated by Nrf2 is NADPH oxidase-4 (NOX4) [41].

GSH participates in disposing of ROS both indirectly and directly by neutralizing H_2_O_2_, O_2_^•−^, and ^•^OH, among others [42,43]. Antioxidant functions performed by GSH are connected with the presence of the –SH group (sulfhydryl group; thiol group) belonging to cysteine residue. It constitutes a source of reducing equivalents to scavenge ROS [44,45]. The antioxidant properties of GSH result from the fact that it serves as a cofactor for the GPx, among others [46]. GPx participates in disposing of H_2_O_2_ and lipid hydroperoxides (LOOH), reducing them to H_2_O and nontoxic lipid alcohols (LOH), respectively [29]. In the course of these reactions, GSH is oxidized to GSSG [47,48]. Generated GSSG is then reduced to GSH by glutathione reductase (GR), which constitutes another source of GSH. NADPH participates in this reaction, which, in turn, is oxidized to NADP+, creating a redox cycle, which prevents distortions of the oxidant–antioxidant balance [29,49]. GSH is also directly used in the detoxification of products of oxidative stress and xenobiotics in reactions catalyzed by glutathione-S-transferase (GST) [33,50]. These are mainly reactions of the elimination of electrophiles, such as 4-hydroxy-2-nonenal, which is the product of lipid peroxidation [24]. GSH may also react with other non-enzymatic antioxidants. It is used, for instance, as a substrate in the reaction of the reduction in oxidized vitamin C [24].

**Figure 1:**
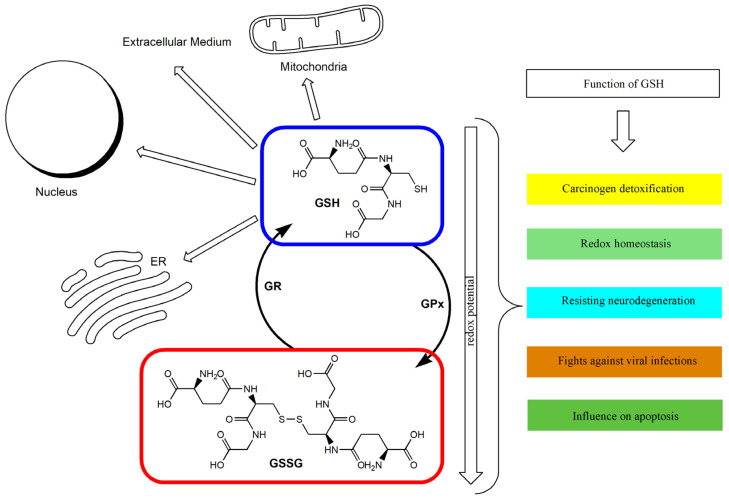
The glutathione redox cycle: transport and functions. ER: Endoplasmic reticulum; GPx: glutathione peroxidase; GR: glutathione reductase; GSH: reduced glutathione; GSSG: oxidized glutathione.

The GSH/GSSG ratio in the cell has been used as an index of cellular redox status [26,51]. Under appropriate conditions, this ratio exceeds 10:1, and its decrease is connected with the increase in oxidative stress in the cell [26,52]. GSH deficiency may therefore play a significant role in the process of aging and in the pathogenesis of numerous diseases [35]. Intracellular GSH depletion leads to cell death by ferroptosis [53]. A distortion in the GSH/GSSG balance toward the oxidizing state activates signaling pathways, leading to a decrease in cell proliferation and the enhancement of apoptosis [54]. It has been proven that a GSH redox imbalance (resulting from deficits of GSH and GSH-related enzymes) may be the primary cause of the many neuropsychiatric and neurodegenerative disorders [55]. In the case of malignant tumors, it has been indicated that GSH metabolism may play both beneficial and pathogenic roles. It has been confirmed that an elevated level of GSH promotes metastasis, which has been shown in liver and melanoma cancer among others [56,57]. At the same time, GSH participates in the removal and detoxification of carcinogens [33,57]. Disturbances in GSH homeostasis have also been indicated in patients with cystic fibrosis, metabolic, cardiovascular, immune, and inflammatory diseases [28,58,59]. GSH depletion and intracellular redox status alterations also accompany certain viral infections [60,61,62]. Figure 1 shows the main routes of glutathione transport and its functions.

The antioxidant defense and regulation of the redox state affect the continuous process of converting the default form of GSH into its oxidized and reduced forms. Acute oxidative stress can reduce the cell’s ability to convert GSSG back into GSH.

## 3. The Role of Glutathione and Pro-Glutathione Compounds in the Immune Response

During viral infection, the sulfur-containing amino acids (methionine and cysteine) may be preferentially used for the synthesis of acute-phase proteins instead of GSH production. The relationships between GSH and immune system functions were previously described [63,64,65,66,67,68,69]. Innate immunity, which is the first line of antiviral defense before the development of adaptive immunity (T cell responses and antibodies), involves the production of ROS and cytokines, which enhance the host response by recruiting and activating other immune cells [67]. Their metabolic activity is dictated by the specific functions and counteracts the redox imbalance caused by the viral production of ROS. For example, in proinflammatory macrophages the pentose phosphate pathway reduces glutathione to ensure cellular antioxidant capacity. Further, the antigen-stimulated T cells proliferate and produce lymphokines being controlled by redox-sensitive transcription factors [70].

There are two types of T cell responses, namely T helper (Th) cell type 1 associated with cellular immunity (production of interleukin IL-12, IL-2, IFN-γ) and Th cell type 2 related to humoral immunity (production of IL-4, IL-5, IL-6, IL-13, and antibodies). It has been demonstrated that the redox potential of GSH, in contrast to antioxidants without sulfhydryl groups (e.g., vitamin C) [71], modulates Th1/Th2 immune response in T cells as well as in the antigen-presenting cells, i.e., macrophages, dendritic cells, and B cells [65,71,72]. Nevertheless, vitamin C was also claimed to participate in the immune response during an early phase of virus infection through the production of type I interferons [73]. The high level of GSH in the antigen-presenting cells correlates with the increased production of IL-12 favoring Th1 response [68], and probably reduces the disulfide bonds of an antigen, which is a crucial step of antigen processing. It also seems that the redox potential of GSH may increase the activity of thiol proteases essential in antigen processing and the cleaving of invariant chains from major histocompatibility complex class II, the main role of which is presenting processed antigens to CD4(+) T-lymphocytes [74,75].

The intracellular and plasma level of GSH has been found to be depleted during infections (such as human immunodeficiency virus (HIV) [76], influenza viruses [77], rhinovirus [78,79], hepatotropic viruses [80,81,82], and HSV-1 [83]) according to mechanisms and kinetics specific to the virus and host cell type [70]. In effect, an oxidative intracellular environment may impair the function of Na^+^/H^+^ antiporters, decreasing the intracellular pH and favoring an early phase of viral replication [84]. In a later phase of viral replication, GSH depletion may be a result of preferential incorporation of cysteine to viral proteins [85]. However, the viral replication can be inhibited by the replenishment of glutathione, preferably in the form of its precursor or ester derivatives. Such an effect may be achieved by administration of *N*-acetyl-*L*-cysteine (NAC), which is a source of thiols for GSH synthesis. Similarly, this goal may be achieved by the supplementation of pro-glutathione compounds, which easily permeate into the cell, e.g., glutathione monoethylester (GSH-OEt), I-152 (which is a codrug of NAC and *S*-acetyl-*β*-mercaptoethylamine), *S*-acetylglutathione, *N*-butanoylglutathione, and GSH-C4 [60,65,70]. It has been confirmed that GSH increases the proliferation of T cells, secretion of IL-2, synthesis and turnover of IL-2 receptors [86,87,88], and the replenishment of intracellular GSH with NAC, which restores IL-2 production through T cells [89]. Moreover, NAC decreases the production of IL-4 and increases the production of IFN-γ supported by the same Th1 predominance [90]. The plasma level of anti-Tat IgG1 (typical of Th2 response) as well as the levels of anti-Tat IgG2a and IgG2b measured by ELISA assay were lowered and increased, respectively, among Tat-immunized mice pretreated with GSH-C4 or I-152, implying the Th1 response [66,70,91]. On the other hand, GSH loss in murine macrophages decreases the secretion of IL-12 and leads to switching Th1-associated cytokines production towards the Th2 immune response [72]. Furthermore, NAC and GSH-OEt enhance the GSH/GSSG ratio and the expression of IL-12 mRNA [92]. For the promotion of viral replication, viruses are able to manipulate the host’s redox-sensitive pathways, such as Nrf2 or nuclear factor kappa-light-chain-enhancer of activated B cells (NF-κB). Nrf2 is a transcription factor that controls the expression level of genes encoding proteins that ensure the intracellular redox homeostasis components of glutathione- and thioredoxin-based antioxidant systems or GST among others [93]. Meanwhile, NF-κB (consisting of c-Rel, RelA (p65), RelB, p50/p105, and p52/p100) together with an inhibitor of NF-κB kinase (IKK) proteins regulates many physiological processes, such as inflammation, innate- and adaptive-immune responses, and cell death [94,95]. The proposed role of GSH molecule to cope with the viral infection could be the inhibition of NF-κB activation. That precludes further signal transduction events facilitating virus (HIV, HSV-1) replication [96,97,98] by ROS scavenging and interference with IKK or with the translocation of NF-κB into the nucleus [70,99]. Additionally, the modification of p50 may inhibit DNA from binding to NF-κB consensus sequences [70].

**Figure 2:**
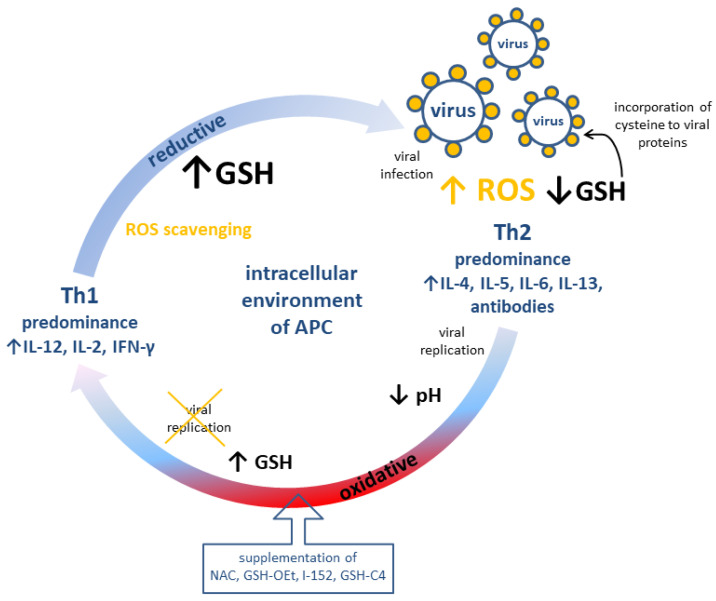
Possible modulation of adaptive immune response (cellular, Th1 and humoral, Th2) by redox potential of GSH in antigen-presenting cells. APC: Antigen presenting cell; GSH: reduced glutathione; GSH-C4: *N*-butanoylglutathione; GSH-OEt: glutathione monoethylester; I-152: explanation in the text; IL: interleukin; NAC: *N*-acetyl-*L*-cysteine; ROS: reactive oxygen species.

The role of GSH and pro-GSH compounds in the immune response is summarized in Figure 2.

## 4. The Alteration of GSH Homeostasis in Viral Hepatitis

Viral hepatitis is a major public health problem in all parts of the world. Acute viral hepatitis (AVH) is a systemic illness that mainly affects the liver. HAV, HBV, HCV, HDV and HEV are the viruses that cause instances of acute viral hepatitis [100,101,102]. A persistent HBV, HCV, HDV or HEV infection can progress to chronic hepatitis (CH), which in turn can evolve into liver cirrhosis (LC), hepatocellular carcinoma (HCC) and other extrahepatic manifestations [100,101,102,103,104]. A significant portion of HBV-infected patients is in the inactive carrier state (ICS). This phase is characterized by normal transaminase levels, little viral replication and minimal liver necroinflammatory activity [105]. HDV can only infect simultaneously with HBV. In the case of HDV and HBV coinfection, most often an acute self-limited hepatitis occurs [100]. HAV and HEV are mainly transmitted via the fecal–oral route, but in the developed countries, hepatitis E is predominantly a zoonosis [100]. HEV infection can cause fulminant hepatitis failure, especially in pregnant women, with a mortality rate of up to 30%, and the virus can be vertically transmitted from infected mothers to their infants [106]. HBV, HCV and HDV infections are transmitted parenterally, mainly as a result of tissue disruption [100,101]. After penetrating into hepatocytes, viruses replicate and then, through a direct cytopathic mechanism or a secondary immune response, lead to hepatocyte damage and necrosis [107,108,109,110,111].

### 4.1. Hepatitis B Virus (HBV)

The HBV virus belongs to the Hepadnaviridae family. It is a partially double-stranded, capsid virus composed of lipids and surface proteins that form an antigen (HBsAg). The viral genome also encodes a number of other important proteins, such as polymerase, regulatory protein X (HBx), and core protein, including its secreted variant HBeAg [112,113,114]. The epidemiology of HBV infections has revealed distinct geographic and ethnic distributions of 10 HBV genotypes (A-J) [113,114]. HBx, HBsAg, and HBV core antigens are described as viral proteins that participate in ROS formation. HBV infection is able to change the levels of antioxidant enzymes in an Nrf2-/antioxidant response element (ARE)-dependent and -independent fashion [115]. Increased Nrf2-activation may either impair virus elimination or prevent the elimination of an HBV-associated hepatocellular carcinoma [112].

ROS are useful for HBV assembly via heat shock protein-90 (HSP-90) interaction with the core protein [26,115]. HSP-90 facilitates HBV capsid assembly, which is an important step for the packing of viral particles [116]. Kim et al. [116] suggest that the low GSH and high ROS level following an HBV infection may cause HBV capsid assembly to be facilitated. The presence of GSH changes HSP-90 conformation, abrogating HBV capsid assembly by 42%. The levels of GSH are lower in patients with chronic hepatitis than those in healthy individuals [117,118,119,120,121,122,123,124,125]. An observation of markedly enhanced oxidative injury in infected patients with chronic HBV infection was made, which is not only related to a decrease in the levels of GSH but also to significantly increased levels of GSSG [120]. Tsai et al. [123] have reported the redox status in patients with HBV-associated hepatocellular carcinoma who had been HBV carriers for 20 years. This study showed a significantly higher increase in the GSSG level, and a decrease in the GSH level and the ratio of GSH/GSSG in the study group when compared to the controls. Alavian et al. [126] suggest that this implies that compensatory mechanisms, which are mostly active at the beginning of the disease, start to fail as the disease course progresses. Therefore, findings within GSH levels may change as the disease progresses and becomes more severe and/or chronic. Severi et al. [127] have shown that induction of HBV replication did not influence the total level of GSH in the cells, but the GSSG/GSH total ratio has been observed to triple after 72 h of HBV induction, indicating that GSH depletion occurred in favor of its oxidized form. In the GSH redox cycle, antioxidant enzymes also play a significant role in maintaining the oxidative balance in the cell.

Controversies have been seen in GPx activity. For example, some researchers have reported decreased activity of GPx [119,120,124], while another study found an increase [128], or no significant changes [123]. The decline in GSH level may lead to a decrease in enzyme activity [122]. Abel et al. [125] observed reduced GPx activity in the hepatocellular carcinoma tissue. They stated that the lower enzyme activity in the HBV-infected carcinoma tissue resulted from a further disruption in the antioxidant defense system (double insults of hepatocellular carcinoma and HBV infection). Shaban et al. [122] showed that GPx levels were decreased significantly when compared to the control in the following sequence: HCC < LC < CH < AVH < ICS. The levels of hepatic GSH were severely depleted, where the GR activity was enhanced in patients in the inactive carrier state, and in the acute viral hepatitis, chronic hepatitis, liver cirrhosis and hepatocellular carcinoma groups when compared to the control group. Studies have also shown increased activity of GR in erythrocyte in acute viral hepatitis, chronic hepatitis [129] and in the blood of patients with HBV infection in hepatocellular carcinoma [123]. Table 1 summarizes studies that reveal the glutathione status in patients with viral hepatitis.

### 4.2. Hepatitis C Virus (HCV)

HCV is a small-enveloped virus with one single-stranded positive-sense RNA in the *Flaviviridae* family. The structural proteins (Core, E1, and E2) are essential components of the HCV virions, whereas the non-structural proteins (p7, NS2, NS3, NS4A, NS4B, NS5A and NS5B) are not associated with virions but are involved in RNA replication and virion morphogenesis [147]. Structural core and nonstructural protein 5A (NS5A) are considered to be the main activators that induce mitochondrial dysfunction and NOXs expression in hepatocytes, producing large amounts of ROS and LOOH [147,148]. The replication of HCV takes place in the membrane compartment associated with the cellular endoplasmic reticulum [147]. This virus is classified into seven different genotypes, which affects the response to different HCV treatments and the progression of liver failure [149,150]. Genotypes 1, 2, 3, 4 and 6 are further subdivided into a series of subtypes [150]. Oxidative stress contributes to insulin and interferon resistance, disorders of iron metabolism, liver cirrhosis and hepatocellular carcinoma [151]. Liver iron accumulation is associated with HCV infection [152]. An increase in serum ferritin has been described in patients with chronic hepatitis [138].

HCV-core protein and HCV-nonstructural protein induce different antioxidant defense response in hepatocytes [142,151,153]. As a result of the expression of the HCV core protein, hepatocyte cell lines experience increased oxidative stress and reduced GSH levels. At the same time, the overexpression of the NS5A protein causes an increase in GSH [142]. It was determined that the acute phase of HCV infection is characterized by an increase in the level of ROS, while the chronic phase, on the contrary, is characterized by a decrease in the level of ROS. An agent that can inhibit HCV replication may be Nrf2. This protein was called an oxidative stress factor. It induces the expression of cytoprotective genes, e.g., inhibits replication by upregulating hemeoxygenase-1 [43,147,148,154].

In the early phase of HCV infection, ROS-induced mitogen-activated protein kinase/c-Jun N-terminal kinase (JNK) or protein kinase C signaling mediates the activation of Nrf2- and Nrf2-activated antioxidant genes [115]. The acute HCV infection is associated with an early induction of proteins functioning in cellular stress responses, including the Nrf2-mediated oxidative stress response [43]. Carvajal-Yepes et al. [141] have reported an inhibitory effect on Nrf2 signaling due to the binding of Maf proteins to NS3, an integral part of the replication complex. In HCV-positive cells, the virus interferes with the Nrf2/ARE signaling pathway, resulting in a lack of activation of cytoprotective genes, elevating ROS levels and autophagy, and thus releasing HCV particles [155]. In constitutively infected cell lines to replicate HCV RNA (Huh-7.5 cells), GSH levels remain constant throughout the first 5 days of infection points to a sufficient reductive capacity of the infected cell in the early stage of infection [140]. In the early phase of the disease, which occurs between the 2nd week and the 6th month after infection, infected cells can activate Nrf2, which provides protection against oxidative stress. Then, when the activity of Nrf2 becomes unfavorable for the virus, i.e., in the later phase of infection, the activity of this factor decreases [153]. Increased iron storage is believed to contribute to increased production of oxygen free radicals and a significant reduction in glutathione (GSH) levels. This reduction is associated with an increased percentage of oxidized glutathione (GSSG), indicating increased GSH turnover [138]. According to research by Lima et al. [132], the rate of viral replication affects GSH production. In the acute phase of infection, HCV replicates rapidly, leading to extensive apoptosis in infected cells [43]. This phase is characterized by pronounced oxidative stress, caused primarily by the activity of NOX4, accompanied by a reduced level of GSH and a reduced GSH/GSSG ratio [43]. Conversely, chronic HCV infection is characterized by lower levels of both HCV replication and apoptosis. ROS production returned to baseline, GSH levels were replenished and the GSH/GSSG ratio increased [43]. Roe et al. [156] report a significant rise of GSSG and a significant decrease in the levels of glutathione metabolites, *S*-methylglutathione and cysteinylglycine, in the HCV-infected human hepatoma cell line Huh-7.5. However, the increased GSH concentration has been demonstrated by de Mochel et al. [157] using the same in vitro infection system. It is suggested that the elevation of GSH occurs in the transient adaptation to sublethal oxidative stress. In contrast to the research of de Mochel et al. [157], several clinical studies reported the decreased hepatic GSH content in HCV chronically infected, untreated, chronic HCV infected patients, which was found both in serum, plasma and hepatic tissue [117,120,133,135,138,139] (Table 1). The increased activity of γ-glutamylcysteine ligase limits the rate of de novo GSH synthesis [80]. Antioxidant drugs have also been shown to increase GSH levels and therefore counteract the progression of liver diseases [135].

NS5A is identified as the viral protein that mediates glutathione peroxidase 4 (GPx4) induction in a phosphatidylinositol-3-kinase-dependent manner pathway activation, protecting HCV-infected cells against lipid peroxidation [140]. GSH is used by GPx4 to reduce LOOH to its alcohol form, which is a crucial step in preventing ferroptosis. The effect of the downregulation of Nrf2 is the inhibition of GPx4, in which case LOOH accumulates, worsening the damage and causing ferroptosis [158]. Studies show that the activity of GPx in erythrocytes and serum is lower in patients with chronic hepatitis than in the healthy control group [159,160]. GPx activity is also reduced in the tumor tissue compared to the control liver tissue [161]. It is considered that decreases in selenium and GPx levels may participate in the impairment of an antioxidant defense and may trigger the process of hepatic fibrosis [131,160]. On the other hand, Ismail et al. [137] and Yalcin et al. [134] reported increased GPx activity in patients with chronic hepatitis and liver cirrhosis, respectively. The GPx concentration was higher in the blood of patients responding to the treatment [128,131,159]. Czeczot et al. [161] and Villegas et al. [162] observed an increase in GR activity in liver cirrhosis and hepatocellular carcinoma compared to the control tissue. Villegas et al. [162] have drawn the conclusion that GR is an essential enzyme involved in the reduction in oxidative stress produced during liver transplantation among cirrhotic patients.

HCV genotypes exhibit varying abilities to induce oxidative stress. Among patients with chronic hepatitis, diminished glutathione (GSH) content was observed in both serum and liver tissue. Specifically, patients with genotype 1b displayed a significant decrease in glutathione levels in the liver, plasma, and lymphocytes when compared to patients with genotypes 2a/2c and 3a, as well as the control group [138]. Ansari et al. [133] observed that genotypes 3a and 1a/b have the highest and lowest values of GSH, respectively. However, it should be noted that patients infected with diverse HCV genotypes had lower GSH levels in serum than the healthy controls. GSSG level was significantly different in diverse HCV genotypes, the highest being in the infected patients with genotype 1a/b.

### 4.3. Hepatitis E Virus (HEV) and Hepatitis A Virus (HAV)

HEV is a single-stranded RNA virus that belongs to the *Hepeviridae* family [102]. HEV is classified into eight major genotypes, where genotypes 1 and 2 infect only humans. In the developed countries, hepatitis E is predominantly a zoonosis caused by genotypes 3 and 4 [106]. One of the factors promoting viral replication by affecting regulatory elements is the increase in the level of sex steroid hormones, which occurs during pregnancy. HEV infection aggravates oxidative stress during pregnancy [163]. Lower GSH levels are observed in pregnant women with an HEV infection having preterm delivery and low birthweight newborns compared to healthy pregnant women [143,144]. It is suggested that the GSH level may be used for risk stratification in HEV infection during pregnancy [163].

HAV is a small, nonenveloped RNA-virus of the *Picornaviridae* family [102]. HAV replicates very slowly and does not cause apparent cytopathic effects in cell culture. In old patients with chronic hepatitis, at the beginning of the disease, a marked depletion of GSH is observed [117]. Additionally, Cemek et al. [145] and Popovic-Dragonjic et al. [146] found decreased GSH levels in children with chronic hepatitis.

In conclusion, hepatitis can be caused by various etiological agents, including HBV, HCV, HEV, and HAV. Depending on the etiological factor, the disease can vary in terms of clinical course and rate of progression. In summary, our results demonstrate that pathological processes associated with infection by hepatotropic viruses are accompanied by a lower GSH level and an increase in GSSG level. Data on the activity of GSH-dependent enzymes are still limited and contradictory.

## 5. Glutathione S-Transferase (α-GST, GST-A) as a Marker of Hepatocellular Damage

Detoxicant enzymes, such as GST, metabolize toxic electrophiles and are also considered to be secondary antioxidant enzymes [122]. These enzyme in cells are located in microsomes, mitochondria, and in the cytoplasm. In hepatocytes, the largest amounts are found in the cytoplasm [164]. GST isoenzymes are found in the liver, nervous system, heart, pancreas, kidneys, spleen, lungs, testes, placenta and erythrocytes [164,165]. GST flows out of the hepatocyte cytoplasm when the cell is damaged [166,167]. GST regulates enzymatic antioxidants and performs protective functions against free radicals.

The level of GST decreases significantly in the cytoplasm during toxic damage to hepatocytes. In the initial period of hepatotoxicity action, an increase in the amount of GST is observed, which causes the activation of the Nrf2, which in turn is activated in response to stress, and a cascade of antioxidant factors is activated, such as superoxide dismutase, heme oxygenase, GPx, GST and NF-κB. In addition, Nrf2 regulates the expression of GST, GPx and GR genes [167,168]. GST has an inhibitory effect on the JNK and NF-κB factor. The interaction between macrophage-like cells and also between the liver and nitric oxide synthase (iNOS) has been proven. The increase in the amount of GST during toxic damage causes a decrease in the amount of iNOS in immunocompetent cells [169].

In the case of acute liver injury, an increase in GST was observed on the first day, whereas a decrease was observed three days after the cessation of hepatocyte toxicity [170]. A greater increase in GST than alanine aminotransferase (ALT) and aspartate aminotransferase (AST) was observed in patients with toxic liver damage. Valeeva et al. [171] assessed defective alleles of GST in patients with acute toxic liver damage and chronic hepatitis. It has been proven to be useful in identifying patients at risk of rapid conversion to liver cirrhosis [171]. The increase in GST was assessed in patients with drug-induced liver injury as a consequence of anesthesia. It was proven that the degree of increase in GST during surgical procedures with anesthesia was dependent on the gene’s polymorphism. Patients with the GSTA1*A allele have been shown to be more susceptible to drug-induced damage [166]. GST was measured in patients with drug-induced liver damage caused by cisplatin and paracetamol. In addition to GST, IL-6 and IL-10 were measured, and their levels also significantly increased [172]. Changes in GST in the course of drug-induced liver failure also correlate with an increase in skeletal-muscle-specific mi-RNA: miR-122 and miR-192 [173]. In a double-blind randomized study reported by Zhang et al. [174], GST was measured in patients with ischemic hepatic failure. The highest amounts were noted in patients with obstruction, and prompt reconstructive intervention of vena resulted in a significant and rapid decrease in plasma GST. However, if the decline did not occur after 24 h, GST concentration correlated with a poor prognosis and was associated with the detection of progressive hepatocyte necrosis [174]. There was an increase in GST activity or concentration in patients with hepatic steatosis. It was estimated that the increase in the concentration of GST slowed down the progression to non-alcoholic fatty liver disease [175]. The level of GST in patients was measured to assess liver rejection after transplantation [176]. The growth of the GST concentration was rapid, but a better correlation and growth rate was noted for the interleukin-2 receptor. GST has been also proven to be an important marker of biliary epithelial cell injury, autoimmune hepatitis and de novo autoimmune hepatitis after transplantation [176].

In patients with chronic HBV, HCV infections, and acute infection caused by hepatitis A, B and C viruses, there was an increase in GST, and GST increased faster than ALT and AST [165,177], and values correlated with the degree of hepatocyte damage [124,135,165,177,178]. There are conflicting results concerning GST activity. Shaban et al. [122] presents studies that reveal the depression of GST activity in different stages of HBV infection (Table 1). They concluded that the decrease in the level of GSH and the activity of GST indicated that the liver had a lower ability to detoxify the ROS, resulting in an increased availability of potentially carcinogenic molecules.

GST levels can be an indicator of ongoing liver damage because of its low molecular weight, uniform hepatic distribution, high cytosolic concentration, and short half-life (the plasma turn-over is 60 to 90 min) [170,174,176,177,178,179].

## 6. The Potential of *N*-Acetyl-*L*-Cysteine (NAC) and Pro-GSH Molecules in the Viral Infection Treatment

The immune response of the body plays a significant role in liver damage during hepatitis infection [180,181]. HBV and HCV can activate the immune system, leading to the production of tumor necrosis factor α (TNF-α), which induces the synthesis of significant amounts of IL-6 and IL-8 [182,183,184,185,186]. Consequently, this can promote hepatitis and damage liver cells. Viral liver diseases are associated with failure to maintain normal GSH levels. In patients with chronic hepatitis C, the presence of systemic GSH depletion may be a factor underlying resistance to interferon therapy [80]. The limitation in the use of GSH as a therapeutic agent is its unfavorable biochemical and pharmacokinetic properties, such as short lifetime in human plasma and difficulty in penetrating cell membranes [187]. However, Qian et al. [22] showed a link between the therapeutic effect of glutathione on chronic hepatitis B. Intravenous GSH (1200 mg) was effective in lowering serum transaminases, TNF-α, IL-6, and IL-8 compared to the control group. Thus, treatment with GSH can improve liver function and suppress inflammation and liver fibrosis in HBV infections. Jabeen et al. [188] conducted in vitro studies on HBV-infected cell lines HepAD38 and HepG2/pHBV1.3 treated with NAC, which showed an increase in the expression of FAM26F glycoprotein, IRF3 transcription factor, and interferon beta, and thus the inhibition of viral replication. Analogous inhibition of HBV replication was obtained by Hosel et al. [189] in the study of the effect of NAC on HBV-infected HepG2 and HepG2-H1.3 cell lines. It turned out that under the influence of NAC (an ROS inhibitor), there was a decrease in the activity of the signal transducer and activator of transcription 3 protein (STAT3). This protein is activated by proinflammatory cytokines, i.e., IL-6 or TNF-a in response to viral infection, which increases viral replication, and reduces apoptosis and oxidative stress. Waris et al. [190] had previously come to similar conclusions. In in vitro studies on the HBV-infected HepG2 cell line, they showed that antioxidants, such as NAC and pyrrolidine dithiocarbamate, similarly to GSH, inhibit STAT3. In a study by Weiss et al. [191], a 50-fold reduction in viral DNA was achieved within 48 h of the HBV-infected cell line HepG2-4A5 being treated with NAC. In a study on the role of the mitochondrial protein SIRT3 in oxidative stress during HBV infection, it was shown that overexpression of this protein leads to a decrease in the level of ROS. In addition, NAC was shown to inhibit HBV replication in an in vitro study on HepG2.2.15 and HepAD38 cell lines [192].

In the untreated HCV patients, the depletion of GSH levels is restored after treatment with an antioxidant or a combination of antioxidants containing vitamin E, C, and zinc. In addition, signs of improved GPx activity have also been shown after antioxidant supplementation [135]. It seems that the methods of increasing GSH levels also include the use of compounds that promote the availability of cysteine, such as NAC, methionine, *S*-adenosyl-*L*-methionine, *L*-2-oxothiazolidine-4-carboxylate, alpha-lipoic acid, pyrrolidine dithocarbamate, and GSH ethyl ester [190,193,194,195,196,197,198]. In clinical trials, it was suggested that in patients with chronic HCV and HBV infection, NAC alone or in combination with other antioxidants significantly affects plasma GSH concentration and GPx activity of erythrocytes [199,200]. It has also been shown that as a result of oxidative stress, the NS5A encoded by the human HCV RNA genome induces the activation of the transcription factors NF-κB and STAT3. NAC and pyrrolidine dithiocarbamate significantly reduced the activation of these NS5A-induced transcription factors [190]. It has been suggested that there is a possible increase in GSH biosynthesis after NAC supplementation, with this increase being possible in lymphocytes without GSH normalization in the serum of HCV patients [81]. The incorporation of cysteine from NAC into GSH can be suppressed by limiting the GSH export from the liver as a result of liver damage during the course of the disease. In addition, interferon-treated patients have not found consistent therapeutic benefits of NAC, which is explained by the pro-oxidative properties of NAC in the presence of metal ions [196,197,198,201]. Pro-oxidant properties have also been demonstrated for pyrrolidine dithiocarbamate due to an increase in the intracellular level of ferrous or cuprous ions [194,195]. It has been suggested that GSH ethyl ester may be more effective than NAC in restoring proper GSH levels in the liver [196,197,198,201]. The analysis of correlations between antioxidants, oxidative stress, and HCV titer is complicated by the fact that observations are made in in vivo and in vitro studies. Differences in these observations may result from changes in HCV titer in vivo and thus from the excessive induction of oxidative stress compared to in vitro studies. The effects of NAC on patients with viral hepatitis caused by serotypes other than B and C were also studied. Patients infected with HAV usually do not receive treatment related to the presence of the virus, but protective drugs for the liver are introduced. In a study of fifty children aged 1 to 17 years, NAC application was shown to lower liver transaminases levels in less time than in the untreated patients. NAC therefore helps protect the liver [202]. These studies confirm previous pilot studies on a small group of 12 patients with HAV [203].

The result of HEV infection may be acute liver failure manifested by hepatic encephalopathy, jaundice, and coagulation disorders, among others. The study by Nabi et al. [204] in a group of 18 patients with HEV showed an improvement in the overall survival in patients with acute liver failure who were administered NAC compared to the control group of patients with acute liver failure caused by factors other than HEV. In addition, the hospital stay was shortened. Mumtaz et al. [205] administered NAC to a group of 24 patients and compared it with a control group of 16 patients who did not receive NAC. A decrease in mortality was observed in patients treated with NAC. It was concluded that not using NAC is a predictor of mortality. The use of NAC is safe, increases survival time, and is additionally recommended for use in centers without the possibility of performing liver transplants.

Since orally administered GSH is considered to be digested in the human gastrointestinal tract and unable to pass through cell membranes or cross the blood–brain barrier efficiently, it is not therapeutically useful. Instead, non-toxic cysteine precursors can be utilized to increase GSH levels. Several articles have demonstrated the clinical application of NAC in viral liver diseases. Oral NAC has proven to be successful as a drug in patients with viral hepatitis A who develop liver failure and a fulminant form of hepatic encephalopathy [203]. NAC is also used in clinical practice for dengue-fever-associated hepatitis and as a therapeutic agent in patients with asymptomatic HIV, hepatitis B virus, and hepatitis C virus infection, including cases of drug-induced liver injury [206,207].

## 7. Conclusions

Increased ROS levels are thought to play a significant role in the pathogenesis of liver failure. The evaluation of oxidative stress in samples can be exploited as important tools in the assessment of disease status in humans. Multiple mechanisms may contribute to systemic changes of GSH levels in hepatitis virus infection. During infection with hepatotropic viruses, the long-term production of Il-6, IL-8, and TNF-α leads to the generation of free radicals. This cycle of high production of free radicals can lead to GSH depletion. It is not only the inflammatory process via cytokine activation that is important in disease progression, but also cytopathic effects of viruses. For example, in genotype 3 HCV infection, hepatic steatosis can result from the direct viral cytopathic effect. In the works on glutathione status during HCV infection, it has been suggested that genotype 1b has greater intrinsic cytopathy action and may activate a more intense inflammatory process. It was noticed that more severe oxidative stress might be associated with more serious disease in HCV genetic subtype 1a/1b in contrast to subtype 3a. However, the association between glutathione status and background genotyping has been described only in few studies. To our knowledge, unlike in the case of primary hepatotro0pic viruses, there are no data concerning the effects of non-hepatotropic viruses on liver dysfunction associated with glutathione status. In most cases of HBV, HCV, HAV, and HEV infection, we will see lower GSH levels in patients with liver diseases, which is associated with the decreased production and decreased influx of GSH from the liver. In in vitro studies, contradictory data have been reported about the effect of HCV on intracellular GSH metabolism. The decrease in the GSH level as well as the increase in the GSSG level and GR activity are consistently revealed in most of the research. GSH and GSSG levels help monitor the progression and treatment of liver failure associated with these viral infections. More research is needed to determine the precise dependence between GPx activity and HBV/HCV/HAV/HEV. According to the available literature, the measurement of increased levels of GST could be considered as a useful confirmatory test for hepatocellular damage. In conclusion, GSH, GSSG and GST levels, and GPx, GR activity may be considered as useful biomarkers for determining the progression of liver damage in infections with hepatotropic viruses.

## Figures and Tables

**Table 1:** Selected results of glutathione status in patients with the most common types of viral hepatitis.

Name of Virus/Genome	Patients	Sample Site	Parameters		Refs.
			Compared to HC	Additional Analysis/Information	
Hepatitis B virus (HBV)/DNA	n_1_ = 69 CHB n_2_ = 20 HC	Erythrocyte	↓ level of GSH ↓ activity of GPx	-	[119]
	n_1_ = 50 CHB n_2_ = 50 HC		↓ level of GSH	-	[130]
	n_1_ = 54 HCC n_2_ = 57 HC		↓ level of GSH ↓ ratio of GSH/GSSG ↑ level of GSSG No statistically significant changes compared to GPx ↑ activity of GR	-	[123]
	n_1_ = 50 CHB n_2_ = 50 CHC n_3_ = 50 HC		↑ activity of GPx	↑ activity of GPx in CHC patients	[128]
	n_1_ = 132 CHB n_2_ = 195 HC n_3_ = 114 CHC	Plasma	↓ level of GSH in CHB and CHC ↑ level of GSSG ↓ activity of GPx	↑ level of GSSG in CHB patients compared to HCV infection patients ↓ activity of GPx in CHB patients compared to HCV infection patients	[120]
	n_1_ = 30 AHB n_2_ = 10 CHB n_3_ = 25 HC		↓ level of GSH in CHB ↓ level of GSH at the beginning of AHB	The normalization of GSH level of GSH after 10 weeks in AHB	[117]
	n_1_ = 54 CHB n_2_ = 31 LC n_3_ = 20 HC		↓ level of GSH in CHB and LC ↑ level of GST in CHB and LC ↓ activity of GPx in CHB and LC	-	[124]
	n_1_ = 54 CHB n_2_ = 51 IHBC n_3_ = 109 HC	Serum	↓ level of GSH in CHB and IHBC	-	[118]
	n_1_ = 78 CHB n_2_ = 67 NCBI n_3_ = 53 AHB n_4_ = 65 LC n_5_ = 60 HCC n_6_ = 150 HC		↓ level of GSH in the following sequence: HCC < LC < CHB < AHB < NCBI ↓ activity of GST in the following sequence: HCC < CHB < LC < AHB < NCBI ↓ activity of GPx in the following sequence: HCC < LC < CHB < AHB < NCBI ↑ activity of GR in the following sequence: HCC > LC > CHB > AHB > NCBI	-	[122]
	n_1_ = 5 HCC including HBV^+^ n_1_ = 8 HCC including HBV^−^	Liver tissue	-	↓ level of GSH in HBV and HBV tissues compared with the corresponding surroundings (↑ GSH level in the HBV-infected surrounding tissue compared with the non-infected surrounding) Level of GSSG had no significant interaction ↓ GPx in HBV tissues compared with the corresponding surroundings ↑ GPx in HBV tissues compared with the corresponding surroundings ↑ GSH/GSSG ratio in HBV tissues compared with the corresponding surroundings ↓GSH/GSSG ratio in HBV tissues compared with the corresponding surroundings GSH was calculated by subtracting GSSG from the tGSH	[125]
	-	HepAD38 cells		significantly ↑ the ratio GSSG/GSH total in HepAD38 cells after induction of HBV replication	[127]
Hepatitis C virus (HCV)/RNA	n_1_ = 19 CHC n_2_ = 28 HC	Erythrocyte	↓ activity of GPx	↑ activity of GPx in CHC patients compared to CHC pretreatment patients Therapy pegylated interferon alfa-2b and ribavirin	[131]
	n_1_ = 24 CHC n_2_ = 27 HC	Plasma	No correlation between level of GSH or GPx activity and the histological diagnosis, serum ALT, AST, or HCV RNA level	-	[132]
	n_1_ = 5 AHC n_2_ = 37 CHC n_3_ = 25 HC		↓ level of GSH	-	[117]
	n_1_ = 160 CHC n_2_ = 160 HC	Serum	↓ level of GSH in patients infected with genotypes 1a/b, 4, 2a/c, 2b and 3a (group 3a highest and 1a/b lowest values, respectively) ↑ level of GSSG GSSG level was significantly different in diverse HCV genotypes Patients with infection genotype 1a/b had the highest levels of serum GSSG	-	[133]
	n_1_ = 18 CHC n_2_ = 18 HC		↑ activity of GPx	-	[134]
	n_1_ = 9 VHC without antiviral treatment n_2_ = 11 VHC with antiviral treatment n_3_ = 12 HC	Whole blood	↓ level of GSH in patients with VHC (n_1_, n_2_) before the antioxidant supplementation ↑ levels of GSH after the supplementation ↑ activity of GPx in patients with VHC (n_1_, n_2_) before the antioxidant supplementation ↓ activity of GPx in patients with VHC (n_2_) after the antioxidant supplementation ↑ activity of GR in patients with VHC (n_2_) before the antioxidant supplementation, which were ↓ after the supplementation ↑ activity of GST in patients from n_2_ before the antioxidant supplementation	Treatment—pegylated interferon combined with ribavirin Supplementation (vitamin E 800 mg, C 500 mg and zinc 40 mg) ↑ activity of GST in patients with n_2_ before the antioxidant intervention compared to patients with n_1_	[135]
	n_1_ = 49 CHC n_2_ = 59 HC	Peripheral blood mononuclear cells	Absence of statistical significance levels of GSH and GSSG, similar activity of GPx and GR	↑ GSH synthetic capacity in patients with CHC, especially in those who did not respond to IFN therapy	[136]
	n_1_ = 21 CHC n_2_ = 7 HC	Liver tissue	↑ activity of GPx	-	[137]
	n_1_ = 130 CHC n_2_ = 23 HC	Liver tissue, plasma, lymphocytes	↓ level of GSH	-	[138]
	n_1_ =75 CHC n_2_ = 22 HC		↓ level of GSH	-	[139]
	-	Huh-7.5 cells	-	↑ in vitro level of GPx4 mRNA in CHC patients compared with control liver biopsies Upon viral eradication, GPx4 transcript levels returned to baseline in vitro and also in the liver of patients	[140]
			-	↑ expression of GPx in the HCV-positive samples from patients with CHC as compared with the control	[141]
			-	↓ GSH level content in infected cells in the acute phase of HCV infection (The GSH accompanied by high rates of viral replication and apoptotic cell death) ↑ GSH level content in infected cells in the chronic phase of HCV infection The GSH content was obtained by subtracting the GSSG from the tGSH	[43]
		Huh-7.5 cells HepG2-derived C34 and E47 cell lines	-	↓ GSH level correlated with the level of HCV core protein expressed ↑ GSSG level correlated with the level of HCV core protein expressed ↑ GSH level correlated with the of HCV-NS proteins expressed The GSH content was obtained by subtracting the GSSG from the tGSH	[142]
Hepatitis E virus (HEV)/RNA	n_1_ = 30 P-AVH n_2_ = 30 NP-AVH n_3_ = 30 CH (pregnant women)	Serum	↓ level of GSH	-	[143]
	n_1_ = 59 AVH n_2_ = 17 FHF n_3_ = 303 HC		↓ level of GSH	↓ level of GSH are lower in the FHF cases compared to AVH cases	[144]
Hepatitis A virus (HAV)/RNA	n_1_ = 17 AHA n_2_ = 25 HC	Plasma	↓ level of GSH	-	[117]
	n_1_ = 19 AHA n_2_ = 29 HC	Whole blood	↓ level of GSH	-	[145]
	n_1_ = 50 AHA n_2_ = 50 HC		↓ level of GSH	-	[146]

Abbreviations: AHA—acute hepatitis A; AHB—acute hepatitis B; AHC—acute hepatitis C; AVH—acute viral hepatitis; FHF—fulminant hepatic failure; GPx—glutathione peroxidase; GR—glutathione reductase; GSH—reduced glutathione; GSSG—oxidized GSH; GST—glutathione S-transferase; HC—healthy controls (patients without known liver disease); HCC—hepatocellular carcinoma; IHBC—inactive hepatitis B carriers; LC—liver cirrhosis; NCBI—normal carriers HBV infection; NP.-AVH—non-pregnant hepatitis-E-positive jaundiced women; P-AVH—jaundiced women in the third trimester of pregnancy with HEV infection; tGSH—total intracellular glutathione (including GSH and GSSG); VHC—viral hepatitis C.

## Abbreviations

ALT	Alanine aminotransferase EC 2.6.1.2
AST	Aspartate aminotransferase EC 2.6.1
AVH	Acute viral hepatitis
CH	Chronic hepatitis
GPx	Glutathione peroxidase EC 1.11.1.9
GR	Glutathione reductase EC 1.6.4.2
GSH	Reduced glutathione
GSH-C4	*N*-butanoylglutathione
GSH-OEt	Glutathione monoethylester
GSSG	Oxidized glutathione, glutathione disulfide
GST	Glutathione-S-transferase EC 2.5.1.18
HAV	Hepatitis A virus
HBsAg	HBV surface antigen
HBV	Hepatitis B virus
HBx	HBV X protein
HCC	Hepatocellular carcinoma
HCV	Hepatitis C virus
HDV	Hepatitis D virus
HEV	Hepatitis E virus
HIV	Human immunodeficiency virus
HSP-90	Heat shock protein-90
HSV-1	Herpes simplex virus-1
ICS	Inactive carrier state
IKK	Inhibitor of NF-κB kinase
IL	Interleukin
JNK	c-Jun N terminal kinase
LC	Liver cirrhosis
LOOH	Lipid hydroperoxides
NAC	*N*-acetyl-*L*-cysteine
NF-κB	Nuclear factor kappa-light-chain-enhancer of activated B cells
NOX4	NADPH oxidase-4
NOXs	NADPH oxidases
Nrf2	Nuclear factor erythroid 2-related factor 2
NS5A	HCV nonstructural protein 5A
ROS	Reactive oxygen species
Th	T helper
TNF-α	Tumor necrosis factor α
